# Induction of tumor-specific CTL responses using the C-terminal fragment of Viral protein R as cell penetrating peptide

**DOI:** 10.1038/s41598-019-40594-7

**Published:** 2019-03-08

**Authors:** D. A. Gross, C. Leborgne, P. Chappert, C. Masurier, M. Leboeuf, V. Monteilhet, S. Boutin, F. A. Lemonnier, J. Davoust, A. Kichler

**Affiliations:** 1 Genethon, 91002 Evry cedex, France; 2 INSERM U1151, Institut Necker Enfants Malades, CNRS, Faculté de Médecine, Université Paris Descartes, Sorbonne Paris Cité, UMR8253 Paris, France; 3 INSERM, Unité 1016, Institut Cochin, Université Paris Descartes, Sorbonne Paris Cité, 75014 Paris, France; 4 Laboratoire de Conception et Application de Molécules Bioactives UMR7199 CNRS - Université de Strasbourg, Faculté de Pharmacie, 67401 Illkirch, France

## Abstract

The discovery of tumor-associated antigens recognized by T lymphocytes opens the possibility of vaccinating cancer patients with defined antigens. However, one of the major limitation of peptide-based vaccines is the low immunogenicity of antigenic peptides. Interestingly, if these epitopes are directly delivered into the cytoplasm of antigen presenting cells, they can be efficiently presented via the direct MHC class I presentation pathway. To improve antigen entry, one promising approach is the use of cell penetrating peptides (CPPs). However, most studies use a covalent binding of the CPP with the antigen. In the present study, we focused on the C-terminal domain of Vpr which was previously demonstrated to efficiently deliver plasmid DNA into cells. We provide evidence that the peptides Vpr55-91 and Vpr55-82 possess the capacity of delivering proteins and epitopes into cell lines as well as into human primary dendritic cells, without the necessicity for a chemical linkage. Moreover, immunization of HLA-A2 transgenic mice with Vpr55-91 as the sole adjuvant is able to induce antigen-specific cytotoxic T lymphocytes against multiple tumor epitopes.

## Introduction

Synthetic peptides represent an attractive source of antigen to stimulate T cell immunity in animals and humans. They have a number of advantages over whole protein vaccines including improved specificity, safety, ease of manufacture and characterization, and lastly the capacity to perform large-scale synthesis. However, when administered alone, these antigens are in most cases weakly immunogenic by themselves. Despite this, several tumor antigens including gp100 and MAGE-3, loaded on antigen-presenting cells (APCs), have been used in phase I/II clinical trials^1^. APCs loaded with tumor antigens have also been approved by US Federal Food Administration for the treatment of prostate cancer but require autologous cells from the patients and are expensive^1^. To further improve broad application and clinical efficacy of cancer vaccines, there is therefore a crucial need for the development of more efficient peptide/protein-based vaccines.

The problem linked with the absence of adjuvant, is that antigen-specific T cells may recognize the antigen but are improperly activated since APCs are poorly attracted to the site of immunization and remain immature. Thus adjuvants, which are a group of structurally heterogeneous compounds, are used to induce or increase antigen-specific immunity by enhancing the speed, strength and duration of the immune response. Although many different adjuvants have been developed and studied, only very few are approved for human use: aluminum salts; MF59, an oil-in-water emulsion used in flu vaccines; monophosphoryl lipid A in pollen-allergy vaccine and AS04 (MPL + alum) for a human papillomavirus vaccine^2^. The structural requirements of adjuvants are poorly understood. However, it is known that facilitation of antigen transport, uptake and presentation by APCs draining the vaccine injection site is of major importance for the effectiveness of vaccines. This in turn can be achieved by different means such as repeated or prolonged accessibility of antigen at the site of injection and/or increased loading of APCs with antigen^3^. Prolonged antigen maintenance at the injection site is effectively established by oil emulsions for example while nano-and microparticles rather facilitate antigen uptake^4^.

Protein/peptide-based vaccines are processed through the endocytic pathway and are mainly presented via the MHC class II pathway, generating pre-dominantly helper CD4^+^ T cells. CD8^+^ cytotoxic T cells (CTL) can also be induced but require efficient cross-presentation, a mechanism by which exogenous antigens are processed and presented onto the MHC class I molecules of antigen-presenting cells. Among several strategies designed to bypass this limiting presentation step, one promising approach is the use of cell penetrating peptides (CPPs). CPPs such as TAT are cationic peptides able to penetrate efficiently into cells^5–7^. These peptides can be used to deliver into cells molecules of interest such as drugs^8^, proteins^9,10^ and nucleic acids^11,12^. It has been shown that TAT-TRP2 epitope can translocate intracellularly into mature DCs and prolong DCs presentation of MHC–TRP2 peptide complexes to antigen-specific T cells^13^. In addition, the propensity for endosome escape demonstrated by some CPPs can promote enhanced antigen presentation by MHC class I molecules, leading to more efficient CTL responses than the naked antigen pulsing method^14,15^. The two most commonly used methods for designing CPP incorporating immunogenic antigens are i- chemical linking via covalent bonds and ii- coupling achieved via recombinant fusion constructs produced by bacterial expression vectors. These methods however present limitations due to the covalent linkage between carrier and epitope: in particular they are dependent on an efficient degradation and processing pathway for MHC I presentation, and they are not always easy to implement.

**Figure 1:**
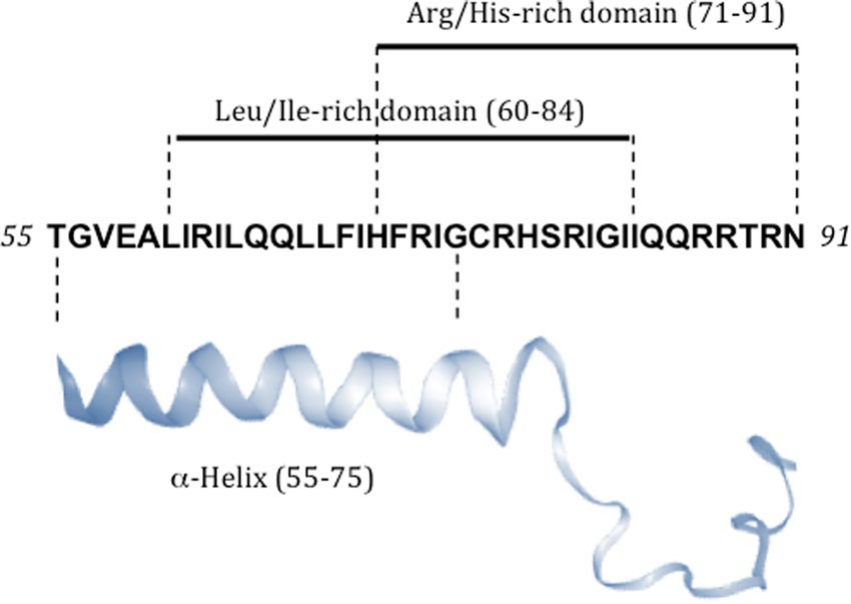
The Vpr55-91 peptide. The C-terminal domain of Vpr is characterized by an α-helix (55–75) and it ends with a flexible C-terminus. This peptide is also characterized by the presence of numerous basic amino acids as well as by presence in the amphipathic helix of a leucine-rich domain. The α-helix cartoon was created from data available in pdb (protein data bank).

We previously reported that the C-terminal domain of Viral protein R (Vpr) - a small accessory protein of 96 amino acids of human immunodeficiency virus type 1 (HIV-1) - is a CPP. Vpr is able to efficiently deliver plasmid DNA into different cell lines^16^ and among the different tested fragments, Vpr55-91 (Fig. 1) was the most efficient one^17^. Moreover, since endocytosis has been revealed as Vpr’s major entry pathway^17^, and efficient expression occurs after delivery of a DNA cargo, it suggests that Vpr peptides possess a permeabilizing activity that allows endosomal escape. Accordingly, using fluorescently labelled versions of the C-terminal domain, it has been shown that the peptide without cargo enters very efficiently into cells^18^.

Based on these properties of Vpr, we explored here its capacity to deliver proteins and epitopes into cells without covalently coupling the carrier peptide to the antigenic cargo. We found that Vpr-derived peptides markedly enhance the delivery of antigens to APCs *in vitro*. In addition, we show that complexes using the C-terminal fragments of Vpr were sufficient to induce antigen-specific CTL responses against multiple epitopes in mice.

## Results

### Efficiency of protein transduction

**Figure 2:**
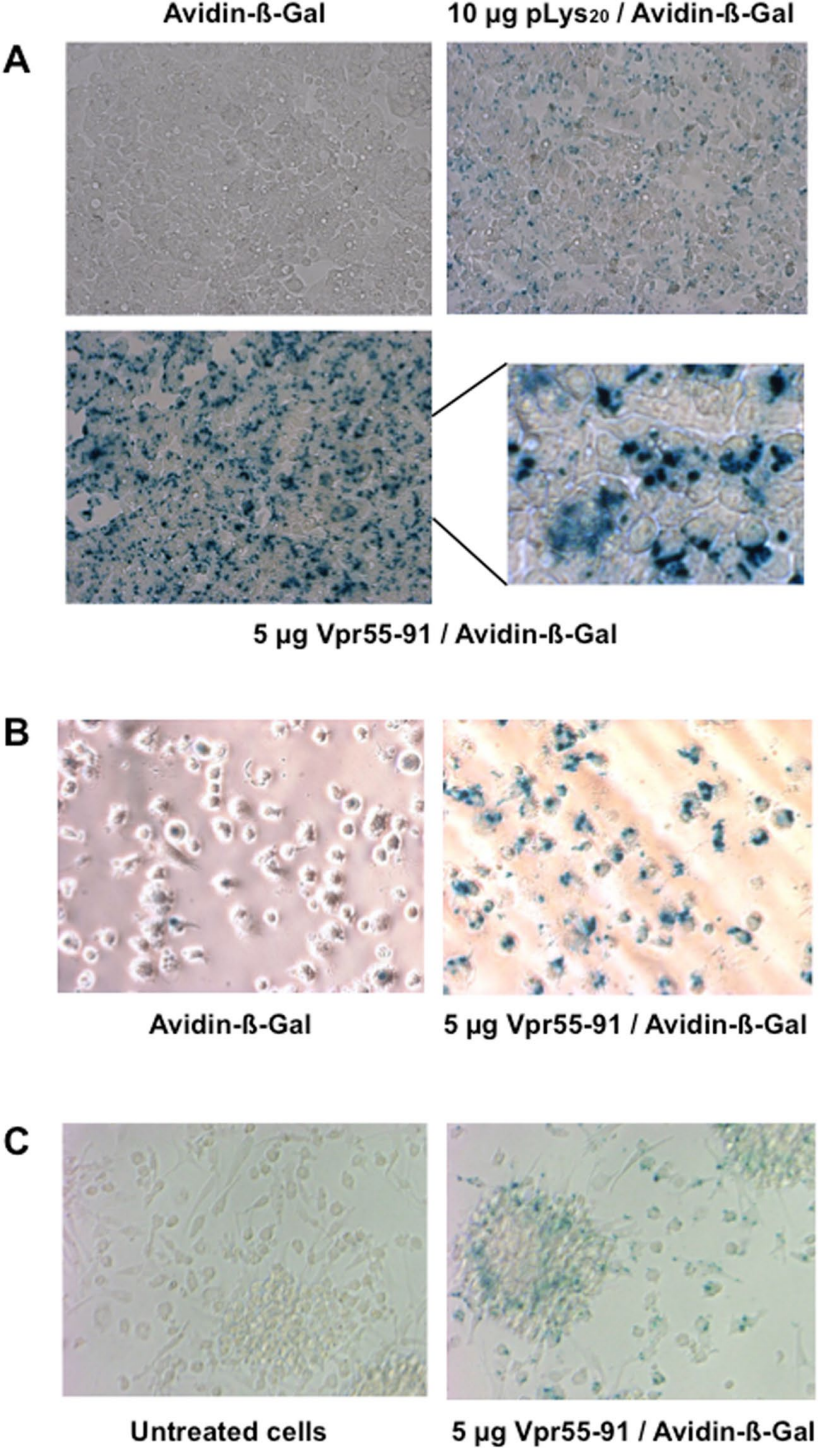
Delivery of an exogenous protein into different cell types. The protein delivery assay was performed using 0.5 µg of avidin-β-Galactosidase per well. The protein was diluted in PBS (50 µL) and mixed or not with 50 µL of PBS containing either 10 µg of pLys_20_ or 5 µg of Vpr55-91. After 30 minutes of incubation at room temperature, serum free culture medium was added (final volume 300 µL) and the mixture was put onto the cells. (**A**) Experiment conducted on HepG2 cells. After 3h30 of incubation at 37 °C, X-Gal staining was performed. The figure shows the results of the staining obtained with cells incubated with avidin-β-Galactosidase, cells incubated with 0.5 µg avidin-β-Gal + 10 µg of pLys_20_ or with 0.5 µg avidin-β-Gal + 5 µg Vpr55-91 (at two different magnifications). (**B**) Experiment conducted on immature human DCs. After 2h30 of incubation at 37 °C, X-Gal staining was performed. (**C**) Experiment conducted on mature human DCs. After 3 h of incubation at 37 °C, X-Gal staining was performed.

We first evaluated the ability of Vpr55-91 peptide (Fig. 1) to deliver a non-covalently linked protein into human cells. HepG2 hepatocytic cells were incubated with 0.5 μg avidin-β-Gal cargo alone or mixed with 5 μg of Vpr55-91, and after 3.5 hours, X-Gal staining was determined. Results show that with Vpr55-91, an important number of HepG2 cells present blue dots in the cytosol while in the absence of Vpr, no or very few cells were positive for β-Gal staining (Fig. 2A). As expected, since β-Gal is delivered as a protein and not as a gene, the cells do not present an homogenous staining. Using a polylysine with a degree of polymerization of around 20 as a control peptide, we observed an increased staining as compared to avidin-β-Gal alone, but still lower than the one obtained with Vpr55-91, indicating that the cationic nature of Vpr is not sufficient by itself for optimal protein delivery.

To confirm these results, similar delivery experiments were conducted on CHO cells using a fluorescently labeled bovine serum albumin. Fluorescence quantification using flow cytometry proved that Vpr55-91 was superior to pLys for BSA-FITC transport (Fig. S1). To demonstrate that Vpr55-91 was not only able to attach the cargo to the cell surface but also to deliver it intracellularly, we made use of trypan blue. Trypan blue is a non-permeant dye that was previously reported to quench the green fluorescence of cell-surface attached yeast particles^19^ as well as fluorescein-CPP^20^. Data showed that dye treatment of cells incubated at 4 °C - when there is no endocytosis - indeed results in complete switch off of the fluorescence that is present extracellularly (Fig. S2A,B). In contrast, when incubated at 37 °C, trypan blue treatment could only reduce to a certain extent the fluorescence proving that a fraction of the fluorescent protein was internalized (Fig. S2C,D).

Next, we investigated whether the Vpr peptide is able to deliver avidin-β-Gal to primary human professional antigen presenting cells. Monocyte-derived CD11c^+^ HLA-DR^+^ human immature dendritic cells (Mo-DC)^21,22^ were incubated with β-Gal protein alone or mixed with Vpr55-91. Again, a significant X-Gal staining was detected when Vpr55-91 was used whereas most untreated cells or cells incubated with Vpr55-91 or avidin-β-Gal alone were negative (Fig. 2B, and data not shown). Notably, no maturation of Mo-DC was observed after incubation with Vpr+/- avidin-β-Gal, as assessed by flow cytometry analysis of CD40, CD83, CD80 and CD86 maturation and costimulatory markers (data not shown). Finally, we looked at mature DCs, which are very efficient to prime naïve T cells but are poorly phagocytic. As shown in Fig. 2C, Mo-DC matured with lipopolysaccharide were also stained after incubation with β-Gal protein mixed with Vpr55-91.

Taken together, our results show that Vpr55-91 is a CPP efficient for delivering proteins into cells in culture, including primary human dendritic cells.

### Tumor-associated antigen delivery

**Figure 3:**
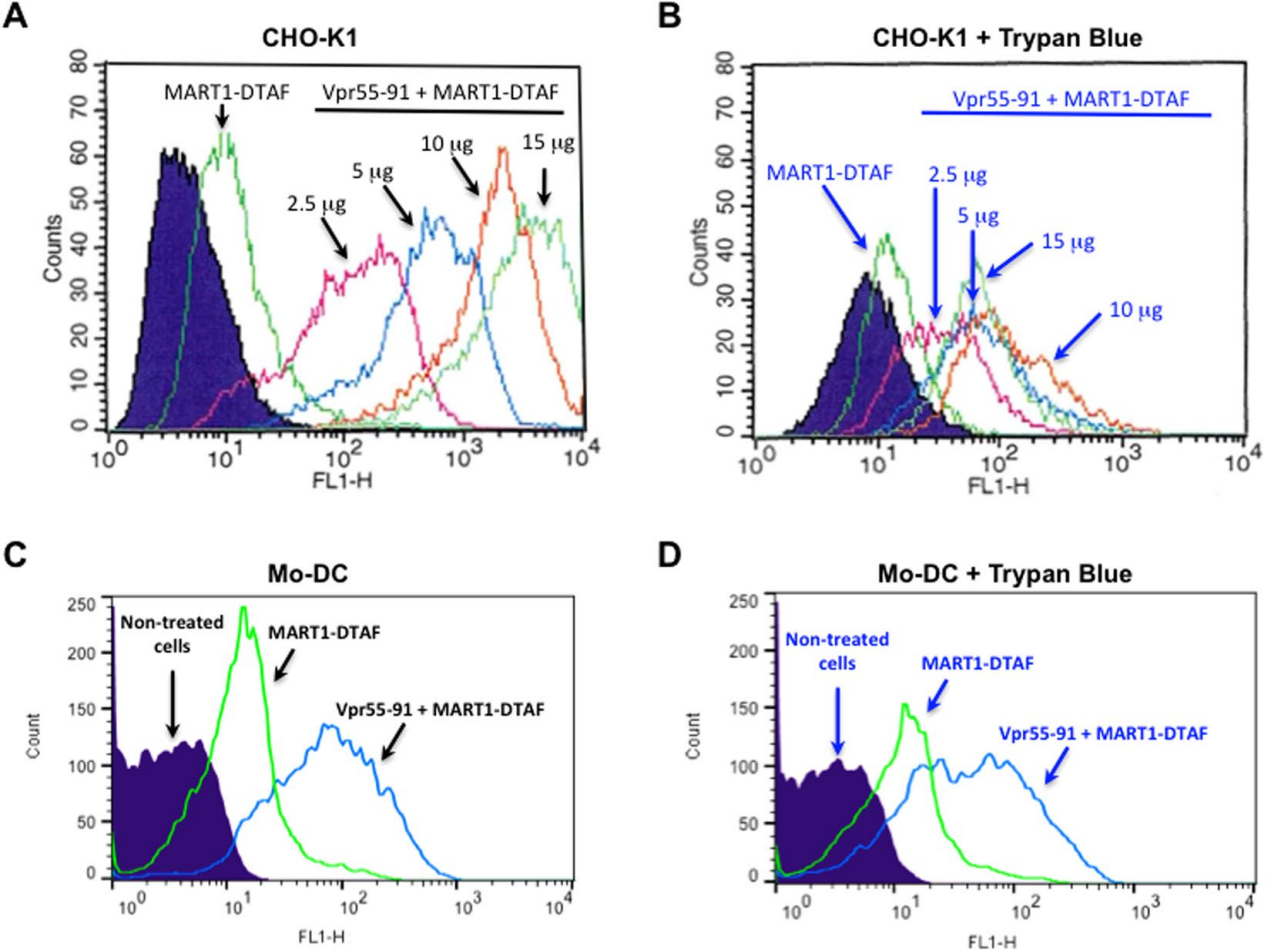
Delivery of a peptide antigen with Vpr55-91. The epitope delivery assay was performed using 5 µg of the fluorescent DTAF-MART-1 epitope per well. The epitope was diluted in PBS (final volume 50 µL) and mixed or not with 50 µL of PBS containing various amounts of Vpr55-91 (2.5; 5; 10 or 15 μg). (**A**) After 30 minutes of incubation of the complexes at room temperature, serum free culture medium was added (final volume 300 µL) and the mixture was put onto the CHO-K1. After 2 h of incubation at 37 °C, cells were washed and analyzed by flow cytometry. Non-treated cells were used as control (purple area). (**B**) Samples analyzed in (**A**) were treated with trypan blue for 10–15 min before flow cytometry re-analysis to quench the fluorescence of cell-surface attached peptides. (**C**) After 30 minutes of incubation at room temperature of Vpr/DTAF-MART-1, AIMV culture medium was added (final volume 500 µL) and the mixture was put onto human dendritic cells. After 3 h of incubation at 37 °C, cells were washed and analyzed by flow cytometry. Non-treated cells were used as control. (**D**) Samples analyzed in (**C**) were treated 15 minutes with trypan blue before flow cytometry re-analysis.

Next, we studied the capacity of the Vpr peptide to deliver a tumor-associated antigen. A fluorescently labelled peptide of the well characterized HLA-A*0201-restricted MART-1 epitope was chosen as a prototypic melanoma antigen (DTAF-MART-1)^23^. HLA-A0201 negative CHO-K1 cells, unable to directly bind this HLA-A0201-restricted epitope onto their own cell surface HLA molecules, were chosen for this assay. CHO-K1 cells were incubated with DTAF-MART-1 with increasing doses of Vpr55-91 or without Vpr55-91 and fluorescence intensity of the cells was measured by flow cytometry. Results indicate that when mixed with Vpr55-91, association of MART-1 epitope with the cell is much more efficient as compared to MART-1 alone (Fig. 3A). We also note a dose-dependent delivery.

Since our goal was to deliver the antigenic epitope into the cytosol to access the processing pathway that leads to presentation on MHC class I molecules, a major concern was to distinguish membrane-bound cargo from those which were internalized. As for the BSA-FITC experiments described above, we used trypan blue. First, we showed that this dye is able to switch off the fluorescence of the DTAF-MART-1 peptide in a dose-dependent manner (Fig. S3). Importantly, when CHO-K1 cells were incubated with MART-1-DTAF plus Vpr55-91, fluorescence was still observed in the presence of trypan blue, indicating that a significant amount of epitope has been internalized (Fig. 3B). In contrast, when the experiment was performed at 4 °C, trypan blue treatment severely reduced the fluorescence (Fig. S4).

Finally, antigenic peptide delivery was assessed in human dendritic cells. Mo-DCs were incubated with either DTAF-MART-1 epitope alone or DTAF-MART-1 epitope mixed with Vpr55-91. As shown, the addition of Vpr55-91 to the labelled epitope very significantly increased the association of DTAF-MART-1 to APCs (Fig. 3C). Again, trypan blue treatment experiments revealed that a significant fraction of the fluorescent peptide was internalized (Fig. 3D). Altogether, these results show that Vpr55-91 is a CPP able to deliver a tumor-associated antigen into the cytoplasm of human cells, in particular into dendritic cells.

### *In vivo* immune response against epitopes associated with Vpr55-91

**Figure 4:**
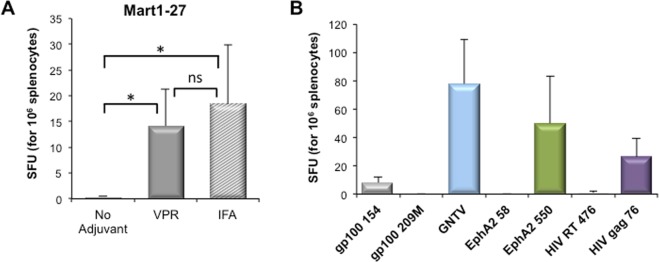
Immunogenicity of peptides associated with Vpr55-91. HLA-A*0201 transgenic mice were injected subcutaneously with 100 µL of indicated epitope and adjuvant, sacrificed at day 7 and their splenocytes tested by an IFN-γ ELISPOT assay. (**A**) Mice were injected with 25 µg of MART-1 epitope, alone (n = 3) or mixed with either IFA (n = 6) or 25 µg of Vpr55-91 (n = 12). Data represent the mean ± SEM and are pooled from three independent experiments. (**B**) Mice were injected with 25 µg of indicated epitope and 25 µg of Vpr55-91 peptide. Data represent the mean ± SEM of 3–6 mice per group pooled from two independent experiments. n.s. not significant, *P < 0.05, ** P < 0.01 (Mann-Whitney).

Immunization with class I restricted epitope alone is poorly effective. Since Vpr55-91 enhances the antigen association and internalization into DCs *in vitro*, we tested whether injection of Vpr/epitope complexes can induce an immune response *in vivo*. HLA-A*0201 transgenic mice were injected subcutaneously with HLA-A*0201-restricted MART-1 epitope, alone or mixed with either incomplete Freund adjuvant (IFA) or Vpr55-91, and immune responses were analyzed 7 days later by an IFN-γ ELISPOT assay. Interestingly, while MART-1 alone was not immunogenic, mixture of MART-1 and Vpr55-91 elicited a T-cell response comparable to that obtained with MART-1 and IFA (Fig. 4A). Next, we evaluated *in vivo* the capacity of Vpr55-91 to confer immunogenicity to a panel of immunodominant tumor and viral HLA-A0201 restricted epitopes. As shown in Fig. 4B, approximately half of the tested epitopes were immunogenic (gp100_154_, GNTV, EphA2_550_ and HIV gag_76_), with frequency of IFN-γ secreting cells ranging between 10 and 80 cells per million splenocytes.

### Covalent linkage between an epitope and the CPP

**Figure 5:**
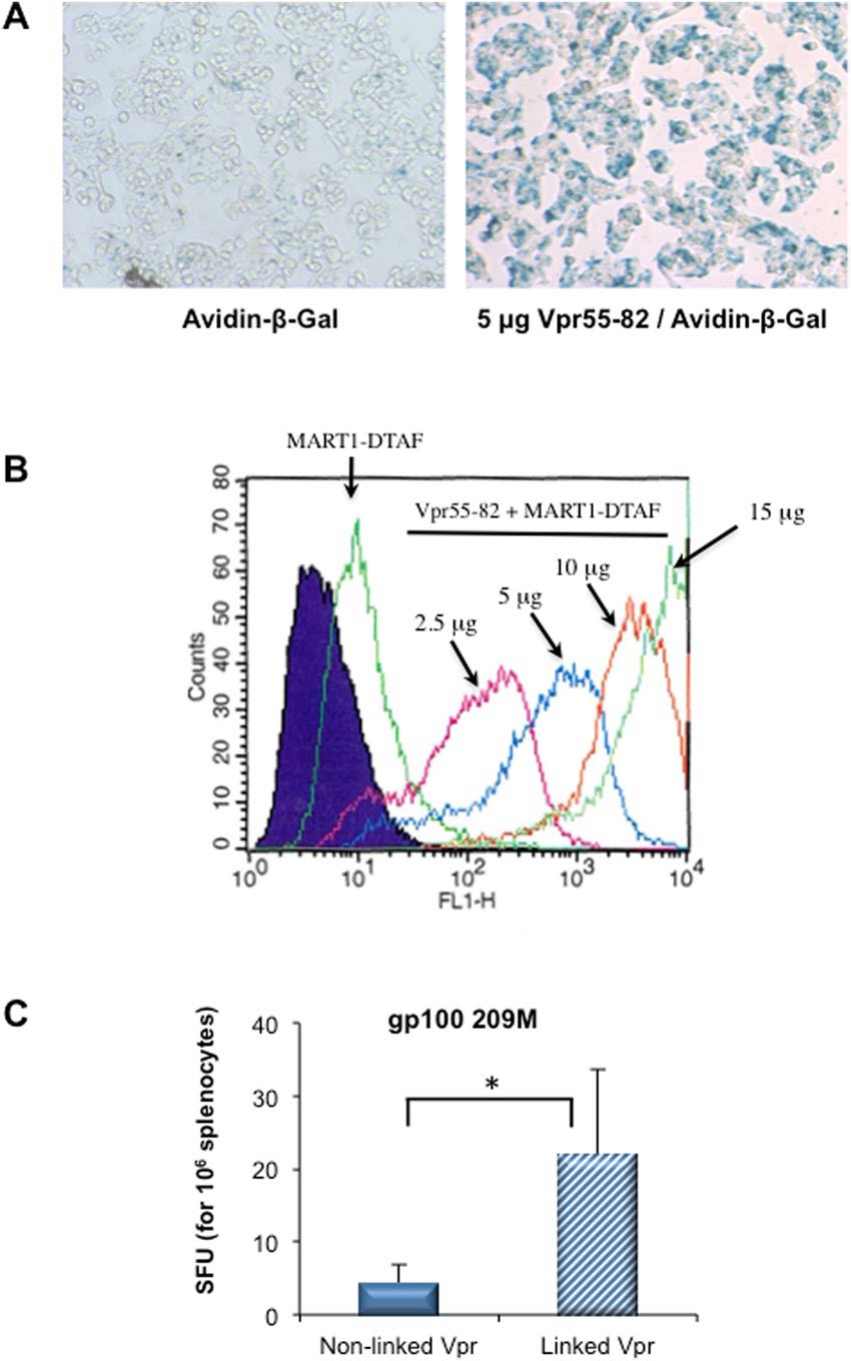
Immunogenicity of the Vpr55-82-gp100 conjugate. (**A**) Protein transduction assay conducted with the CPP Vpr55-82 on HepG2 cells. The experimental conditions were similar to those described in the legend of Fig. 2A,B Epitope transduction assay on CHO-K1 cells performed with Vpr55-82 using the same conditions as those described in Fig. 3(C) HLA-A*0201 transgenic mice were injected subcutaneously with 100 µL of either Vpr55-82 plus gp100_209M_ or a linked Vpr55-82-gp100_209M_ peptide, sacrificed at day 7 and their splenocytes tested by an IFN-γ ELISPOT assay. Data represent the mean ± SEM of 6 mice per group pooled from two independent experiments. *P < 0.05 (Mann-Whitney).

The reasons why certain Vpr/epitopes induce a response while other formulations do not are unclear. We hypothesized that non-responder epitopes such as gp100_209M_, did not efficiently associate with Vpr55-91 and should be covalently linked to the CPP. Since Vpr55-91 is already a long peptide we coupled the antigenic epitope to a shorter Vpr derivative, namely Vp55-82. This latter peptide has previously been shown to deliver DNA into cells^17^. Here, we show that Vpr55-82 has similar protein and peptide transduction properties than Vpr55-91 since it efficiently delivers avidin-β-Gal and DTAF-MART-1 into cells (Fig. 5AB). Mice were immunized with gp100_209M_ mixed with Vpr55-82 or a single gp100_209M_-Vpr55-82 peptide and the immune response analyzed 7 days later. As shown in Fig. 5C, the covalent linkage of the Vpr peptide to the antigenic cargo was able to induce an immune response.

### *In vivo* CTL priming with epitopes associated with Vpr55-91

**Figure 6:**
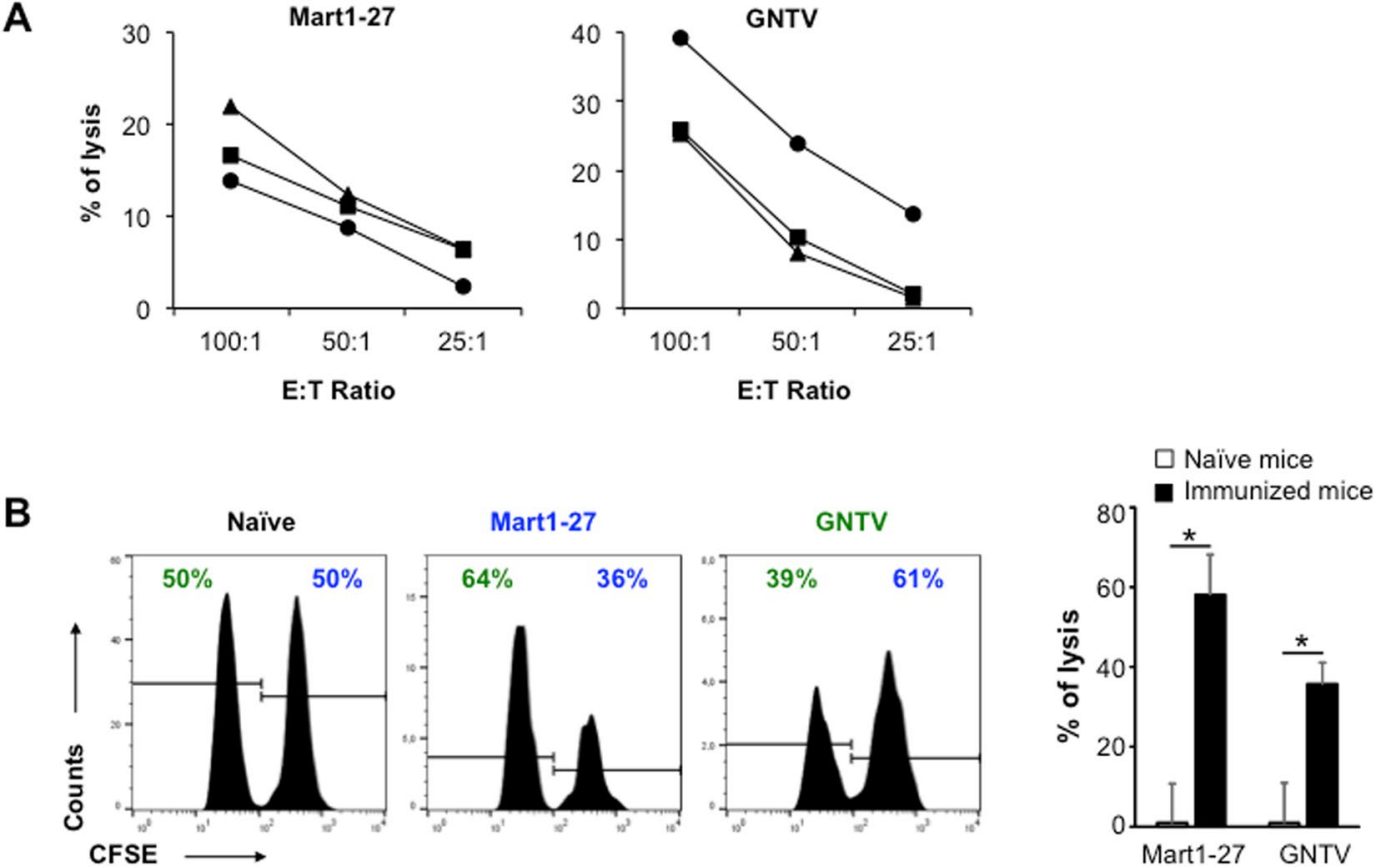
CTL priming with peptides associated with Vpr55-91. HLA-A*0201 transgenic mice were injected subcutaneously with 100 µL of 25 µg of Vpr55-91 mixed with 25 µg of either MART-1 or GNTV epitope, and tested one week later. (**A**) Splenocytes from immunized mice were restimulated *in vitro* and tested against different numbers of ^51^Cr-loaded RMAS-HHD target cells pulsed with one or the other epitope. Each line represents the splenocytes from an individual mouse. (**B**) 10^7^ naïve splenocytes loaded with MART-1 peptide and labelled with 5 µM of CFSE (CFSE^high^) were mixed with 10^7^ naïve splenocytes loaded GNTV peptide and labelled with 0.5 µM of CFSE (CFSE^low^) and were injected into naïve or immunized mice. Flow cytometry histograms show the relative percentage of each of the CFSE targets in naïve, MART-1 and GNTV vaccinated mice two days later. Data represent the mean ± SEM of 4 samples per group (two organs/mice pooled from two different mice) *P < 0.05 (Mann-Whitney).

We next investigated the cytotoxic capacity of the induced CD8^+^ T cells. First, HLA-A*0201 transgenic mice were injected subcutaneously with Vpr55-91 mixed with either MART-1 or GNTV epitope, and one week later, their splenocytes were restimulated *in vitro* to perform a classical ^51^Cr release cytotoxic assay. As shown in Fig. 6A, both epitopes primed cytotoxic T cells able to lyse target cells in an antigen-specific manner. To further confirm these *in vitro* results, we evaluated their *in vivo* cytolytic capacity. Mice were again vaccinated with either MART-1 or GNTV with Vpr55-91, or not immunized (naïve), and 7 days later, equal numbers of differentially CFSE-labelled splenocytes loaded with either MART-1 or GNTV were injected intravenously. Two days later, mice were sacrificed and their spleen and blood were collected to quantify the remaining CFSE-labelled target populations. As shown in Fig. 6B, while both GNTV- and MART-1-pulsed/labelled splenocytes remained in naïve mice (50% of each population), MART-1 vaccinated mice specifically lysed CFSE^high^ MART-1-pulsed splenocytes and GNTV vaccinated mice specifically lysed CFSE^low^ GNTV-pulsed splenocytes. Altogether, these results showed that the association of Vpr derived peptides with different epitope is necessary and sufficient to induce an antigen-specific CTL response in mice.

## Discussion

The concept of protein or epitope delivery directly into the cytoplasm of APCs is attractive since antigens that access the cytoplasm are processed and presented via the MHC class I antigen presentation pathway. In 1965, it was demonstrated that basic amino acid-rich histones and basic poly-amino acids can stimulate the internalization of albumin into tumor cells^24^. Later studies showed that conjugation of poly-L-lysine to albumin enhanced cellular uptake^25^. In 1988, cell membrane penetration of the TAT peptide was observed^6^ and later, many other CPPs have been identified^8^.

In parallel, the discovery of an ever increasing number of tumor-associated antigens^26^ recognized by T lymphocytes opened up the possibility of vaccinating cancer patients with defined antigens. However, one of the major drawbacks of peptide-based vaccines is the low immunogenicity of these epitopes. The ability of CPPs to induce a superior immune response via enhanced delivery and prolonged presentation has been demonstrated with proteins^27^ and peptides^28,29^.

Most studies using CPPs for peptide/protein-based vaccines used a covalent binding of the CPP with the Ag (either through chemical synthesis or recombinant fusion constructs). In the present study we focused on the the C-terminal domain of Vpr which is characterized by an α-helix-(55–75), and it ends with a flexible C-terminus^30^. Of note, this domain is characterized by the presence of numerous basic amino acids as well as by the presence in the amphipathic helix of a leucine-rich domain that contains a short leucine zipper-like motif (Fig. 1). We previously demonstrated that the Vpr derived peptides 55–91 and 55–82 efficiently deliver - through an endocytic mediated pathway - plasmid DNA into various cell lines^16,17^. Release of the DNA probably takes place before acidification of the endosome since the membranolytic activity of the peptides is strongly reduced at acidic pH^17^. This feature made these peptides good candidates for promoting antigen delivery into the cytosol to facilitate their MHC class I presentation, in particular in the case of matured DC which are known to be poorly phagocytic.

Here, we provide evidence that Vpr55-91 and Vpr55-82 possess the capacity of delivering proteins and epitopes into cell lines as well as into human primary DCs. Trypan blue treatment allowed us to prove that the CPP was not only able to attach the peptidic/proteic cargo to the cell-surface but also to allow internalization of a fraction of it. To go one step further into the comprehension of the delivery process we made use of the monensin drug.

Monensin is a proton/sodium ionophore which transports monovalent cations across membranes and thereby equilibrates the external and the internal pH of organelles in intact cells. Since the fluorescence of FITC is pH-dependent (fluorescence decreases significantly when the pH becomes acidic)^31^ it means that if there is an enhancement of the cell fluorescence intensity upon monensin treatment it indicates that fluorescein was localized in an acidic environment (i.e. increase of the fluorescence intensity of the fluorescent marker due to the neutralization of the endosomes)^32^. Figure S5 shows that while there is an increase of the intensity of fluorescence after monensin treatment when cells were incubated with BSA-FITC, pLys/BSA-FITC and pLys/DTAF-MART-1 there was no increase when Vpr was used as transporter (neither with BSA-FITC nor with DTAF-MART-1). These results suggest that Vpr complexes are either in endocytic vesicles that do not acidify or that Vpr allows for a rapid escape - before acidification occurs - of the cargo from the endosomes. This latter hypothesis would fit well with the fact that the membranolytic activity of the Vpr peptide is strongly reduced at acidic pH^17^.

Zhang *et al*. showed that the amphipathic LAH4 peptide^33,34^ was able to facilitate protein internalization and presentation of the TRP-2 tumor antigen, underlining the importance of the endosome and proteasome in this process^35^. Efficiency of vaccines relies on their capacity to activate and deliver antigens into DCs. Here, no maturation of human DCs was observed after incubation with Vpr as assessed by FACS analysis. This result is in agreement with what has been found for others CPPs^36^. Probably the efficient Vpr-mediated delivery of the antigen into the cytosol may protect the peptide from endo-lysosomal degradation and allow a long-lasting antigenic presentation to the T cells, sufficient for T cell priming in absence of strong maturation agents.

Thus, although no mechanism was identified so far for Vpr, a likely explanation relies on an efficient antigen delivery into the cytosol facilitating the access to the MHC class I processing machinery. The absence of chemical linkage between Vpr and the antigenic peptide may prevent trimming and degradation processes required for epitopes chemically linked to CPPs.

Interestingly, in the absence of boost, our vaccine primed CD8^+^ T cells with a single injection and these cells demonstrate antigen-specific cytotoxicity *in vivo*. The frequency of antigen-specific CD8^+^ T cells could appear low as compared with that obtained after viral infection, but is rather high in comparison to other non viral vaccine/synthetic combination. For example, MART-1-27 in the presence of either an helper epitope or incomplete Freund adjuvant, induces a very low CTL response, if any, in HLA-A2 transgenic mice^37^. Efficient vaccination required 2 immunizations or a modification of MART1-27 anchor positions. With the LAH4 cell penetrating peptide, 15 spots per 3 × 10^5^ splenocytes were observed when using the OVA peptide as antigen, whereas it produced 100 spots when CpG adjuvant was added^35^.

The main effector functions of CD8+ T cells are their capacity to secrete cytokines such as IFNγ or TNFα, as well as their capacity to lyse target cells through the coordinated expression of perforine, granzyme and CD107. These functions were assessed using functional assays, rather than using extensive immunophenotyping which are indirectly related to T cell function. Indeed, after *in vitro* antigenic challenge, CD8+ T cells elicited here are able to secrete IFN-γ, as measured by ELISPOT assay (Fig. 4). In addition, we have also shown that these T cells demonstrate cytotoxicity *in vitro* and especially *in vivo*, directly eliminating target cells in an antigen-specific manner (Fig. 6). These results thus indicate that functional effector CD8+ T cells were generated by immunization with Vpr-associated epitope. Interestingly, immune responses are generated here without any apparent need for an adjuvant. This is an important aspect in terms of safety since others CPPs often require adjuvant that are CPP-dependent and have to be precisely defined. For example, efficacy of CTL induction and anti-tumor response of various ZEBRA-derived CPPs variants depend on the combination of Hiltonol, Pam3CSK4 and MPLA adjuvants aiming at activating TLR3, TLR2 or TLR4 respectively^38^. Nevertheless, in the case of the LAH4 CPP, simply adding CpG adjuvant enhanced the response by 5–10 times^35^. DNA sensing by TLR9 occurs into endosomal compartment. Since Vpr peptides are also able to efficiently deliver nucleic acids into cells, probably through endocytosis, one could imagine to add to the Vpr/antigen mixture CpG oligonucleotides for further boosting the immune response.

Finally, another interesting aspect of Vpr-mediated immunization is the diversity of the antigenic peptides able to elicit a CTL response *in vivo* (Fig. 4), as half of the tested tumor and viral epitopes were shown to be immunogenic. Elucidating why only a fraction of the peptides are immunogenic has to be done in order to better define efficient vaccine. This will pave the way for practical multi-epitope immunization which is now effective in the clinic for neoantigen vaccination in cancer patients^39,40^.

## Methods

### Peptides

The Vpr fragments used in this study derive from the protein of HIV-1 strain 89.6 and the sequence of Vpr55-91 is shown in Fig. 1. The peptides Vpr-55–91 and Vpr-55–82 were synthesized by Epytop (France). The MART-1 (Melan-A_26–35_: EAAGIGILTV_-COOH_) peptide synthesized by Epytop (France) was fluorescently labelled using the fluorescein derivative DTAF (5-(4,6-dichlorotriazinyl) aminofluorescein) (DTAF-EAAGIGILTV_-COOH_). The other peptides used in this study are the followings:

Vpr55-82-gp100: TGVEALIRILQQLLFIHFRIGCRHSRIG-IMDQVPFSV_-COOH_

Melan–A_26-35_: EAAGIGILTV_-COOH_

gp100_209M_: IMDQVPFSV_-COOH_

gp100_154_: KTWGQYWQV_-COOH_

GNTV: VLPDVFIRC_-COOH_

EphA2_58_: IMNDMPIYM_-COOH_

EphA2_550_: VLAGVGFFI_-COOH_

HIV RT_476_: ILKEPVHGV_-COOH_

HIV gag_77_: SLYNTVATL_-COOH_

### Cell lines

Dulbecco’s modified Eagle medium (DMEM; Gibco-BRL) was supplemented with 2 mM L-glutamine, 100 units/mL penicillin, 100 µg/mL streptomycin and 10% of fetal calf serum (FCS; HyClone). We used human hepatocarcinoma cells (HepG2 cells) for the protein transduction assay and CHO-K1 (Chinese hamster ovary cells) for the peptide transduction experiments. For the cytotoxic T-cell assay, RMA-S HHD cells, the murine RMA-S lymphoma cell line transfected with the human HLA-A0201 molecule was used^41^.

### Culture of peripheral blood monocytes

Monocytes were generated from samples from healthy volonteers obtained from Etablissement Français du Sang (Reims, France)^22^ in accordance with INSERM ethical guidelines. This method yielded purified (91.9%+/−4.7) CD14^+^CD45^+^ cells as assessed by flow cytometry. Briefly, cryopreserved monocytes were cultured in 6-well plates, for 6 to 7 days, at a density of 1 × 10^6^ cells/mL in AIMV medium (Invitrogen) supplemented with 1% L-glutamine (Invitrogen). Monocytes were differentiated in monocyte-derived dendritic cells (Mo-DC) in presence of 50 ng/mL of recombinant human GM-CSF (Novartis), and 15 ng/mL of recombinant human IL-4 (Tebu-bio). In some experiments, Mo-DC maturation was induced at day 6 by addition of 7 µg/mL of lipopolysaccharide for 24 h (Sigma-Aldrich). Cells were cultured in a humidified incubator at 37 °C and 5% CO_2_.

### Flow cytometric analysis

The Mo-DC phenotype was assessed using three colors immunostaining with biotinylated, phycoerythrin (PE)-, Cy-Chrome (CyC) and allophycocyanin (APC) -conjugated monoclonal anti-CD40 (5C3), anti-CD83 (HB15e), anti-CD80 (L307.4), anti-CD86 (FUN-1), anti-CD11c (B-Ly6), anti-HLA-DR (G46.6) antibodies (all purchased from Becton Dickinson). Data were acquired using a FACSCalibur flow cytometer (Becton Dickinson) and analyzed with CellQuest (Becton Dickinson) or FlowJo software (Tree Star).

### Preparation of Vpr/protein and Vpr/epitope complexes

0.5 µg of avidin-β-Gal and indicated amounts of Vpr peptide or polylysine with a degree of polymerization of ≈20 (Sigma-Aldrich) were each diluted in 25 μL of calcium free PBS and gently mixed. After 30 min of incubation at room temperature, the mixture was diluted with serum-free medium to a final volume of 0.4 mL. The Vpr/epitope complexes were generated using the same protocol as for avidin-β-Gal.

### Protein transduction experiments

240,000 HepG2 cells were plated in 24-well plates one day before the experiment. Before transduction, the cells were rinsed once with serum-free medium. 0.4 mL of culture medium without serum containing the complexes were then transferred into each well and cells were transduced for 3h30 at 37 °C. For Mo-DC, 100,000 cells/well/200 µL AIMV were plated into 48-well plates and 50 µL/well of complexes were added for 2h30–3h at 37 °C. After incubation the medium was removed, cells were washed, fixed and stained with X-Gal. Briefly, cells were washed twice with PBS, fixed in 0.5% glutaraldehyde for 20 min at 4 °C, and washed again. Then, the cells were incubated overnight at 37 °C with X-Gal (5-bromo-4-chloro-3-indolyl-β-D-galactopyranoside). Finally, the cells were washed again twice with PBS before the transduction efficiency was evaluated.

### Flow cytometric quantification of epitope delivery

100,000 CHO-K1 cells/well were plated into 24-well plates and 170,000 monocyte-derived dendritic cells were plated into 48-well plates. The day after, the epitope delivery assay was performed using 5 µg of the fluorescent peptide DTAF-MART-1 per well. The epitope was diluted in PBS (final volume 50 µL) and mixed or not with 50 µL of PBS containing various amounts of Vpr55-91 (2.5; 5; 10 or 15 μg). After 30 minutes of incubation at room temperature, culture medium was added and the mixture was put onto the cells. After 2 to 3 h of incubation at 37 °C, cells were washed and analyzed by flow cytometry (Becton Dickinson FACSCalibur flow cytometer). Non-treated cells were used as control. In order to quench the green fluorescence of cell-surface attached peptides the cells were incubated for 10 min at 4 °C with 0.2% of trypan blue and these cells were then re-analyzed by flow cytometry^19^.

### Ethics statement

Care and manipulation of mice were performed in accordance with national and European legislations on animal experimentation. The experiments were approved by the veterinary office of Essonne, France (Agreement number 91-365). For monocytes, according to French Public Health Law (art L 1121-1-1, art L 1121-1-2), written consent and Institutional Review Board approval are not required for human non-interventional studies.

### Mice immunization and immune response analysis

HLA-A0201/human β2-microglobulin/human CD8α triple transgenic mice^42^ were injected subcutaneously with 100 µL of saline buffer containing 25 µg of antigen alone or mixed with either incomplete Freund adjuvant (IFA) or Vpr55-91 (25 or 50 µg). Immune responses were analyzed 7 days later as previously described^43^.

For IFNγ ELISpot assay, freshly isolated splenocytes (1 × 10^6^/well and serial dilutions) were cultured in Multiscreen nitrocellulose microplates (Millipore) coated with anti-mouse IFNγ Ab with or without 1 µM of indicated epitopes in complete RPMI1640 medium supplemented with 10% fetal calf serum, 2 mM L-glutamine, 50 U/mL penicillin, 50 µg/mL streptomycin and 5 × 10^5^ M 2-mercaptoethanol. For each assay, Concanavalin A was added (5 µg/mL) as a positive control. After 20 hours, spots were revealed and counted using a Bioreader 2000 (BIOSYS, Karben, Germany). Spot forming units (SFU) are represented after subtraction of background values obtained with unpulsed splenocytes (always below 10 spots/well).

For *in vitro* cytotoxic assay, 5 × 10^7^ splenocytes were stimulated *in vitro* with the indicated epitope (10 µM) in complete medium and the bulk responder populations were tested for specific cytotoxicity at day 6 of culture. Briefly, 2.5 × 10^3^ 51Cr-labeled epitope pulsed (37 °C for 60 min) or unpulsed RMA-S HHD target cells were incubated with different concentrations of effector cells in a total volume of 200 µL at 37 °C for 4 hours. After incubation, 100 µL of supernatant was collected and radioactivity was measured in a γ-counter (Wallac). Percentage of specific lysis was determined as: Lysis = (Experimental Release − Spontaneous Release)/(Maximal Release − Spontaneous Release).

For *in vivo* cytotoxic assay, naïve splenocytes loaded with 10 µM of GNTV or MART1-27 epitopes were incubated respectively with 0.5 µM or 5 µM of CFSE (Molecular Probes) at 37 °C for 10 minutes^44^. After washing, the cells were mixed at a 50:50 ratio, and 2.10^7^ cells were injected into the tail vein in 0.2 mL PBS. PBMCs and spleen were collected from individual mice at day 1 and day 2, respectively, labelled with 7-AAD to exclude dead cells and analyzed for CFSE expression by FACS. The percentage of specific lysis of cognate (c) over non-specific/cognate cells (u) was calculated for each immunized mice (i) in comparison of naïve mice (n) as follows: % specific lysis = [(c/u)n − (c/u)i]/[(c/u)n] × 100.

### Statistics

All data are shown as mean ± standard error of the mean (SEM). All statistical analyzes (Mann-Whitney t test) were performed using Prism Software version 6 (GraphPad Software, San Diego, California, USA) and considered statistically significant if P < 0.05.

## Supplementary information

Induction of tumor-specific CTL responses using the C-terminal fragment of Viral protein R as cell penetrating peptide
