# Supplementary material for: Induction of tumor-specific CTL responses using the C-terminal fragment of Viral protein R as cell penetrating peptide
Source: Sci Rep. 2019 Mar 8;9:3937. doi: 10.1038/s41598-019-40594-7 (PMC6408526; doi:10.1038/s41598-019-40594-7)
Supplement: Supplementary file 1 — Induction of tumor-specific CTL responses using the C-terminal fragment of Viral protein R as cell penetrating peptide [file 41598_2019_40594_MOESM1_ESM.pdf]

Induction of tumor-specific CTL responses using the C-terminal fragment of Viral protein R as cell penetrating peptide

D. A. Gross[1,2]*, C. Leborgne[1], P. Chappert[1,2], C. Masurier[1], M. Leboeuf[1], V. Monteilhet[1], S. Boutin[1], F. A. Lemonnier[3], J. Davoust[1,3], A. Kichler[1,4]*

[1] Genethon, 91002 Evry cedex, France; [2] INSERM U1151, Institut Necker Enfants Malades, CNRS, UMR8253, Faculté de Médecine, Université Paris Descartes, Sorbonne Paris Cité, Paris, France ; [3] INSERM, Unité 1016, Institut Cochin, Université Paris Descartes, Sorbonne Paris Cité, 75014 Paris, France; [4] Laboratoire de Conception et Application de Molécules Bioactives UMR7199 CNRS - Université de Strasbourg, Faculté de Pharmacie, 67401 Illkirch, France.

* *Corresponding authors:* david.gross@inserm.fr; kichler@unistra.fr

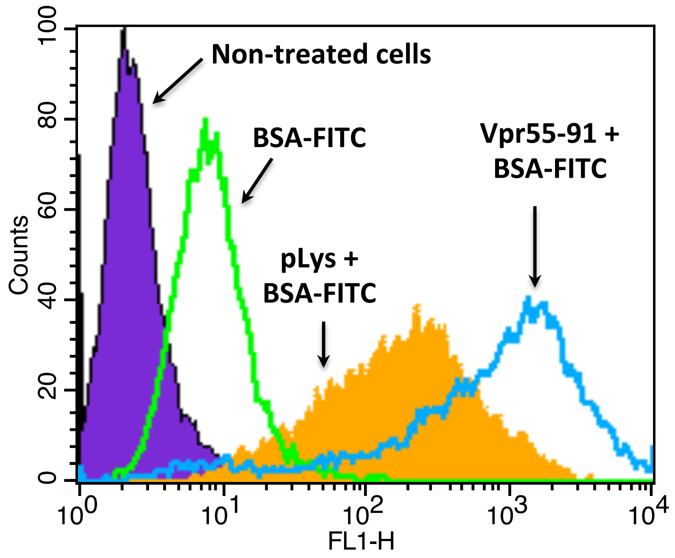

Figure S1. Cell delivery of BSA-FITC performed with either pLys or Vpr55-91.
45,000 CHO cells/well were plated into 24-well plates. Two days after, the protein delivery assay was performed using 4 µg per well of a fluorescently labeled bovine serum albumin (BSA-FITC). The protein was diluted in PBS (50 µL) and mixed or not with 50 µL of PBS containing 4 µg of a pLys with a degree of polymerization of 215 (orange area) or 8 µg of Vpr55-91 (blue curve). After 30 minutes of incubation at room temperature, culture medium was added and the mixture was put onto the cells (300 µL/well). After 3h45 of incubation, cells were washed once with cold PBS, detached and analyzed by flow cytometry. Non-treated cells were used as control (purple area) and the green curve shows the results obtained with BSA-FITC alone.

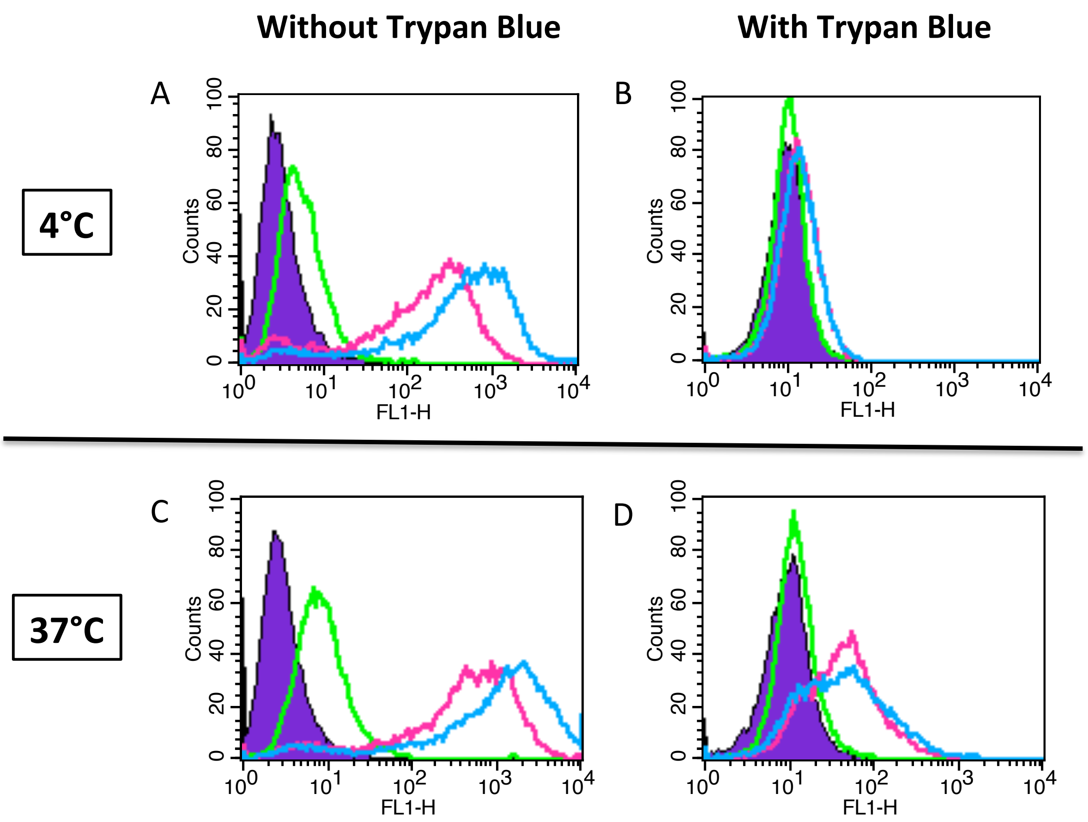

Figure S2. Flow cytometric quantification of BSA-FITC delivery at 4°C and 37°C. 45,000 CHO cells/well were plated into 24-well plates. Two days after, the protein delivery assay was performed using 4 µg of a fluorescently labeled bovine serum albumin (BSA-FITC) per well. The protein was diluted in PBS (final volume 50 µL) and mixed or not with 50 µL of PBS containing 4 µg (pink curve) or 8 µg (blue curve) of Vpr55-91. After 30 minutes of incubation at room temperature, cold culture medium was added and the mixture was put onto the cells (300 µL/well). This experiment was conducted at the same time at 37°C (bottom) and at 4°C (top; in this case the cells were pre-incubated for 30 minutes at 4°C before addition of the complexes). After 2h of incubation, cells were washed once with cold PBS, detached and analyzed by flow cytometry (figures on the left). Non-treated cells were used as control (purple area) and the green curve shows the results obtained with BSA-FITC alone. In order to quench the fluorescence of cell-surface attached peptides the cells were incubated for 15 min at 4°C with 0.2% of trypan blue and these cells were then re-analyzed by flow cytometry (figures on the right).

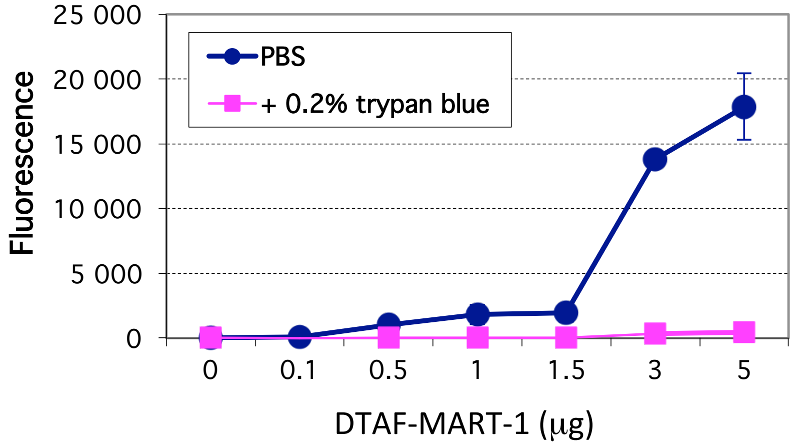

Figure S3. Extinction of the fluorescence using Trypan Blue.

Increasing amounts of DTAF-MART-1 (0.1; 0.5; 1; 1.5; 3 and 5 µg) were diluted with PBS to a final volume of 100 µL. The fluorescence was then recorded using a spectrofluorometer with λexc=490 nm and λEm=515. 100 µL of trypan blue were then added to the wells in such a manner that the final concentration was of 0.2% and the fluorescence of the samples was measured again. The values obtained in wells containing only PBS were considered as background levels.

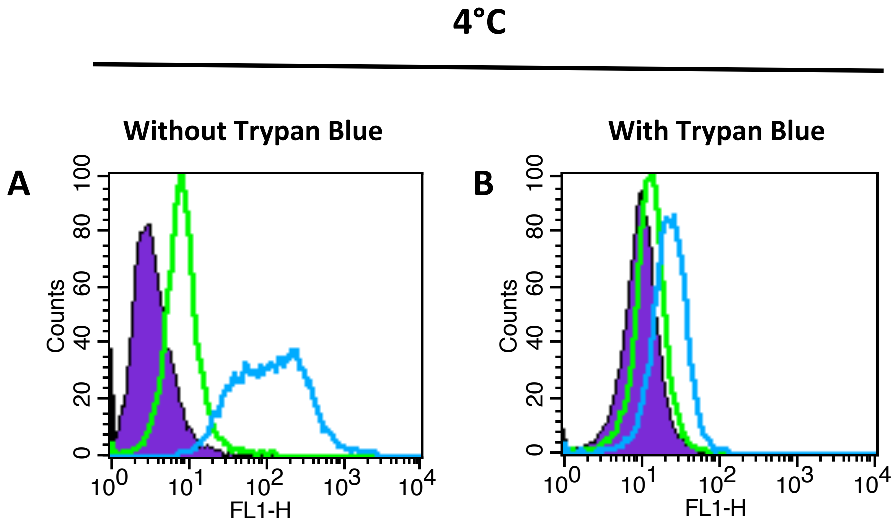

Figure S4. Delivery of a peptide antigen with Vpr55-91 at 4°C.

The epitope delivery assay was performed using 5 µg of the fluorescent DTAF-MART-1 per well. The epitope was diluted in PBS (final volume 50 µL) and mixed or not with 50 µL of

PBS containing 5 μg of Vpr55-91. After 30 minutes of incubation of the complexes at room temperature, cold serum free culture medium was added (final volume 300 μL) and the mixture was put onto the CHO-K1 cells (which were put at 4°C 30 minutes before). After 2h15 of incubation at 4°C, cells were washed once with cold PBS and analyzed by flow cytometry (**A**). Non-treated cells were used as control (purple area), the green curve shows the results obtained with DTAF-MART-1 alone and the blue line corresponds to the Vpr55-91/DTAF-MART-1 mixture. (**B**) Samples analyzed in (**A**) were treated with 0.2% trypan blue for 15 min/4°C before flow cytometry re-analysis.

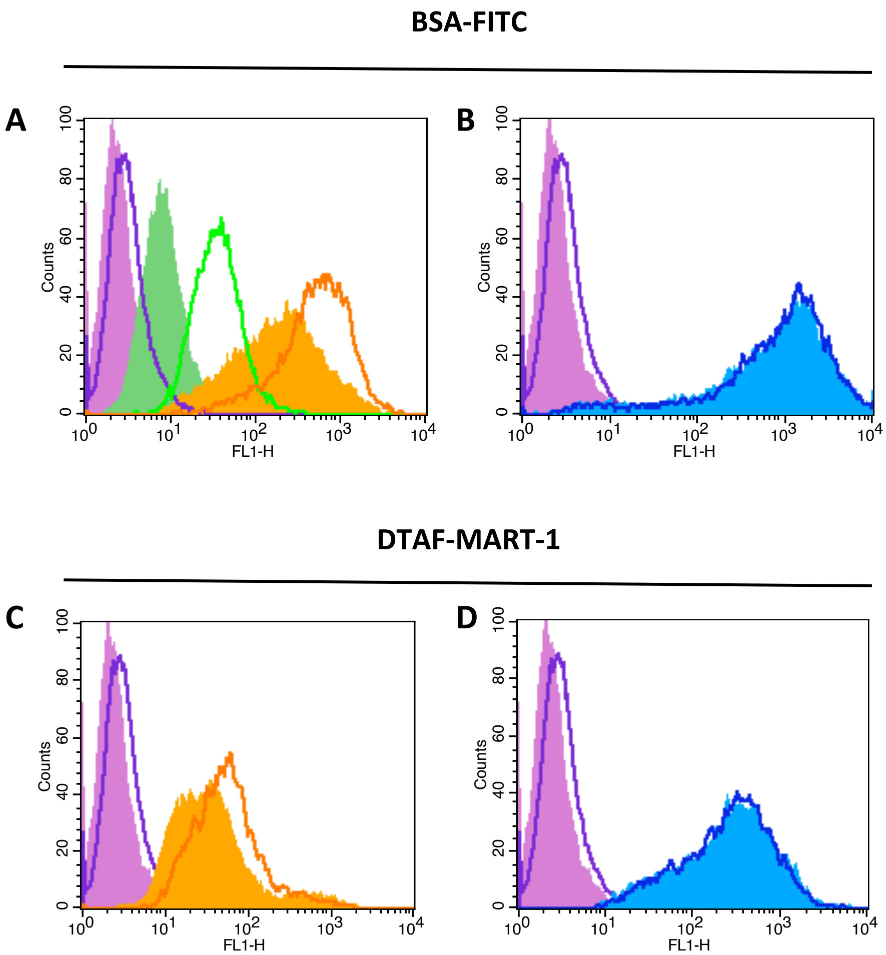

Figure S5. Effect of monensin on the cell-associated fluorescence.

45,000 CHO cells/well were plated into 24-well plates. Two days after, the delivery assay was performed using either 4 μg per well of BSA-FITC (**A, B**) or 5 μg per well of DTAF-MART-1 (**C, D**). The peptide/protein was diluted in PBS (50 μL) and mixed or not with 50 μL of PBS containing a pLys with a degree of polymerization of 215 or Vpr55-91. After 30 minutes of incubation at room temperature, culture medium was added and the mixture was put onto

the cells (300 µL/well). After 3h45 of incubation, cells were washed once with cold PBS, detached and analyzed by flow cytometry (full colored areas). After the first analysis, samples were incubated at 4°C for 30 min in the presence of 50 µM of monensin and then re-analyzed (thick lines). In all figures, non-treated cells are represented by the pink area whereas the control cells treated with monensin are represented by the thick purple line. (**A**) green area: 4 µg BSA-FITC; green line: 4 µg BSA-FITC + monensin; orange area: 4 µg pLys215/4 µg BSA-FITC; orange line: 4 µg pLys215/4 µg BSA-FITC + monensin. (**B**) blue area: 8 µg Vpr55-91/4 µg BSA-FITC; blue line: 8 µg Vpr55-91/4 µg BSA-FITC + monensin. (**C**) orange area: 4 µg pLys215/5 µg DTAF-MART-1; orange line: 4 µg pLys215/5 µg DTAF-MART-1 + monensin. (**D)** blue area: 5 µg Vpr55-91/5 µg DTAF-MART-1; blue line: 5 µg Vpr55-91/5 µg DTAF-MART-1 + monensin.
